# Optimization of the prescription isodose line for Gamma Knife radiosurgery using the shot within shot technique

**DOI:** 10.1186/s13014-017-0919-4

**Published:** 2017-11-25

**Authors:** Perry B. Johnson, Maria I. Monterroso, Fei Yang, Eric Mellon

**Affiliations:** 1 Radiation Oncology / Biomedical Engineering, University of Miami, 1475 NW 12th Ave, Miami, FL 33136 USA; 2 Radiation Oncology, University of Miami, 1475 NW 12th Ave, Miami, FL 33136 USA

**Keywords:** Radiosurgery, Gamma Knife, Gradient index, Optimization, Metastases

## Abstract

**Background:**

This work explores how the choice of prescription isodose line (IDL) affects the dose gradient, target coverage, and treatment time for Gamma Knife radiosurgery when a smaller shot is encompassed within a larger shot at the same stereotactic coordinates (shot within shot technique).

**Methods:**

Beam profiles for the 4, 8, and 16 mm collimator settings were extracted from the treatment planning system and characterized using Gaussian fits. The characterized data were used to create over 10,000 shot within shot configurations by systematically changing collimator weighting and choice of prescription IDL. Each configuration was quantified in terms of the dose gradient, target coverage, and beam-on time. By analyzing these configurations, it was found that there are regions of overlap in target size where a higher prescription IDL provides equivalent dose fall-off to a plan prescribed at the 50% IDL. Furthermore, the data indicate that treatment times within these regions can be reduced by up to 40%. An optimization strategy was devised to realize these gains. The strategy was tested for seven patients treated for 1–4 brain metastases (20 lesions total).

**Results:**

For a single collimator setting, the gradient in the axial plane was steepest when prescribed to the 56–63% (4 mm), 62–70% (8 mm), and 77–84% (16 mm) IDL, respectively. Through utilization of the optimization technique, beam-on time was reduced by more than 15% in 16/20 lesions. The volume of normal brain receiving 12 Gy or above also decreased in many cases, and in only one instance increased by more than 0.5 cm^3^.

**Conclusions:**

This work demonstrates that IDL optimization using the shot within shot technique can reduce treatment times without degrading treatment plan quality.

**Electronic supplementary material:**

The online version of this article (10.1186/s13014-017-0919-4) contains supplementary material, which is available to authorized users.

## Background

A sharp penumbra is a well-known hallmark of Gamma Knife (GK) radiosurgery. When combined with a highly accurate positioning and immobilization system, the modality is capable of treating lesions directly abutting critical structures while minimizing the volume of normal brain receiving high dose. For any radiotherapy device, the width of the penumbra is defined by a variety of factors. Physically, the design of the source and collimation define a geometric component while the energy of the beam along with the composition of the transport medium defines a radiological component. When multiple beams intersect the penumbra is affected by beam overlap, and for certain systems transmission through the tertiary collimator creates a modifying effect. These factors in combination determine the distance between relative isodose lines (IDL) along the target periphery (see Table 1 for description of terms and metrics used throughout the manuscript).

In terms of absolute dose, the penumbra is also affected by the choice of prescription IDL where it is advantageous to prescribe to a line that lies within the dose gradient. For GK based delivery, the 50% IDL is by far the most common selection – largely based on historical precedent and the assumption that prescribing to the 50% IDL provides the steepest dose fall-off outside the target. In previous versions of the Gamma Knife (Model C/4C), this assumption was explored with the authors finding the gradient index optimized at prescription IDLs ranging from 38 to 68% for a single shot plan [1]. The data was presented in a figure with the gradient index placed along the vertical axis and the prescription isodose diameter placed along the horizontal axis. The prescription isodose diameter in the axial plane is governed by collimator size and the choice of prescription IDL. Increasing the collimator size or prescribing to a lower IDL increases the prescription isodose diameter, allowing for the coverage of larger targets. In the aforementioned figure, each of the four collimator sizes available in the previous versions of the GK were plotted by altering the prescription IDL, thus changing the width of the prescription isodose diameter. Interestingly, the plots overlap within certain ranges of isodose (i.e. target) diameters, suggesting that more than one collimator size can adequately conform to a simple target but provide a potentially different gradient index.

While the previous work discussed this point within the context of a single shot plan, the current versions of the Gamma Knife (Perfexion/Icon) provide a fast, automated platform for changing collimator size. This feature enables easy use of the shot within shot technique where two shots having different collimator sizes are assigned the same stereotactic coordinates. By varying the fraction of beam on time allotted for each shot, the width of the prescription isodose diameter can be changed at sub-millimeter increments. In seeking a plan that optimizes both target coverage and conformity, the use of the shot within shot technique greatly expands the solution space. This is due to the fact that shot weighting can be combined with the selection of the prescription IDL in order to best match the width of the prescription isodose diameter with the width of the target. For simple targets, each solution represents a unique plan that achieves the appropriate target coverage and conformity, but may vary in terms of the steepness of the dose gradient and the amount of beam-on time. The relationship between these latter two aspects is currently unknown and represents a type of optimization yet to be fully explored for GK radiosurgery.

In this study these questions are answered through the comparison of over 10,000 shot within shot configurations, each created from profiles extracted from the planning system and characterized using Gaussian fits. The beam configurations fill a multi-dimensional solution space parameterized according to the dose gradient, prescription isodose diameter, prescription IDL, and beam-on time. A strategy is presented for utilizing this space to optimize the planning process. The objective of the optimization is to reduce treatment time while maintaining acceptable plan quality. The optimization relies upon the use of the shot within shot technique to treat small to medium sized lesions using prescription IDLs other than the 50% IDL. The strategy is demonstrated for a number of real cases representing patients treated for metastatic disease in the brain.

## Methods

### Beam configurations

Data was collected using Leksell Gamma Plan version 10.0 configured for the Gamma Knife Perfexion (Elekta Instruments, Stockholm, Sweden). The Perfexion has three collimator settings (4, 8, and 16 mm) which can be set independently for any one of eight sectors. For this work, only uniform sector settings were applied. In order to generate treatment plans, a digital phantom was created using 80 mm for all measurements associated with the skull scaling instrument. The phantom mimicked the shape of a human head with a depth of 8 cm to the center. For each collimator setting, a single shot plan was generated and visualized in the three principal axes. Dosimetric profiles in the left/right (X which is symmetric with Y) and superior/inferior (Z) directions were manually extracted using the line measurement tool. The tool provided the stereotactic coordinates and relative IDL of any point selected along the line. Roughly 70 data points were sampled from each profile and imported into the curve fitting toolbox available within Matlab R2014b (MathWorks, Natick, MA). The data was fit using a combination of Gaussian curves (see Additional file 1: Figure S1).

With each profile parameterized, a series of scripts were written to quantify the prescription isodose diameter and the dose gradient. For the purposes of this work, the gradient was defined as the distance between two relative isodose lines. The first line was always represented by the prescription IDL which could range from 40 to 90% depending on how the plan was prescribed. The second line was selected by multiplying the prescription IDL by a factor ranging from 0.2–0.9. As an example, if the plan was prescribed to the 50% IDL the gradient could be quantified as the distance between the 50% IDL and the 25% IDL (factor = 0.5) or the 50% IDL and the 10% IDL (factor = 0.2). This definition is convenient in that when translated to absolute dose, the gradient distance is calculated between the same dose levels regardless how the plan is prescribed. Using a factor of 0.5 and a prescription dose of 20 Gy, the gradient distance will always be calculated to the 10 Gy IDL.

Using this definition, the gradient distance was tabulated for 153 different beam configurations representing each collimator setting (3 in total) prescribed at IDLs ranging from 40 to 90% (51 in total). In order to expand the work to include the shot within shot technique, the parameterized data was combined to create composite profiles. Variation in beam on time was achieved by using a weighted average to combine the data from two different collimator settings. The weighting was changed at increments of 0.01 which provided 201 unique combinations of the 4/8 mm and 8/16 mm collimator settings. Each combination was prescribed at IDLs ranging from 40 to 90% which increased the overall number of beam configurations to 10,251 (51 IDLs X 201 collimator settings). The composite profiles were validated by comparing the calculated prescription isodose diameter and gradient distance (factor = 0.5) with measurements made directly in the planning system.

### Optimization strategy

A strategy was developed to optimize the selection of collimator weighting and prescription IDL when utilizing the shot within shot technique. The goal was to minimize beam-on time while maintaining an acceptable dose gradient. As 50% is the standard choice for the prescription IDL, the gradient distance (factor = 0.5) for plans prescribed in this manner was set as the baseline for acceptability. Given a new target, the first step was to create a plan utilizing the shot within shot technique prescribed at the 50% IDL (SS_50%_). The collimator weighting was balanced such that the prescription isodose diameter closely matched the width of the target in the axial dimensions. In the next step, a script was written to search the 10,000+ shot within shot configurations for all plans that produced a prescription isodose diameter in phantom that matched to within 50 μm of the same metric when prescribed at the 50% IDL. From these plans the data was culled, selecting only those plans that produced a gradient distance (factor = 0.5) in the axial dimensions which was no more than 3% greater than that produced by the 50% IDL plan. The final step was to choose from this list the plan with the minimum beam-on time (SS_opt_). This strategy was tested for a number of institutional review board approved patients who were previously treated at the local institution for metastatic disease in the brain using the Gamma Knife Perfexion.

## Results

**Fig. 1:**
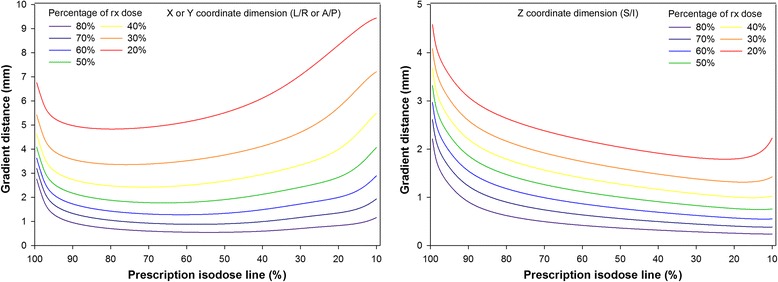
Dose gradient for the X/Y (left figure) and Z (right figure) dimensions, 8 mm collimator setting

Verification of the data parameterization was performed for 18 different shot within shot configurations. The percent difference between manual and calculated metrics (prescription isodose diameter and gradient distance w/ factor = 0.5) was less than 3% (see Additional file 2: Profile validation for complete results). This agreement is very good considering that manual measurements made within Gamma Plan can only be recorded to the nearest tenth of a millimeter. Utilizing the parameterized data, Fig. 1 illustrates the dose gradient for the 8 mm collimator setting. The data are plotted according to prescription IDL where the colored lines represent the gradient distance when calculated using different factors ranging from 0.2–0.8. The figures show the gradient steepest in the superior/inferior direction where it is constantly decreasing and minimized at extremely low prescription IDLs. For the axial dimensions, the prescription IDL which provides the steepest dose gradient depends upon how the gradient is defined. Using a factor of 0.5, the shortest gradient distance for a single 8 mm shot occurs when prescribed somewhere between the 62–70% IDLs. For the 4 mm and 16 mm collimator settings these ranges were 56–63% and 77–84%, respectively. When utilizing the shot within shot technique, the shape of these curves can change drastically with the optimal prescription IDL fluctuating between 40% (the lowest calculated for this study) and 84%. Additional figures highlighting these findings are provided in the Additional file 1: Figures S2 – S4.

**Fig. 2:**
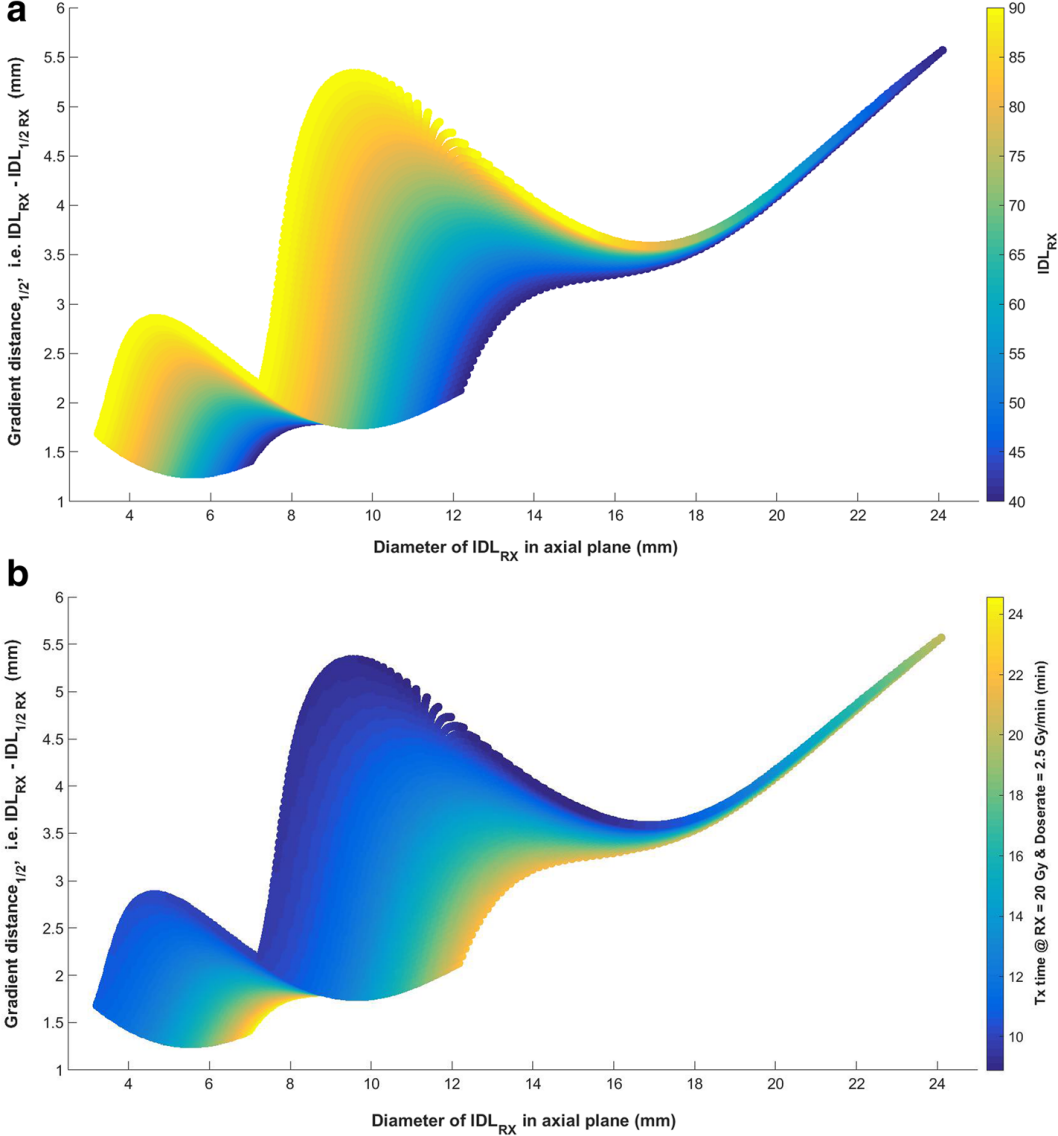
Graphical representation of the optimization space provided by the 10,000+ beam configurations using the shot within shot technique plotted with colors indicating (**a**) prescription IDL or (**b**) treatment time

**Fig. 3:**
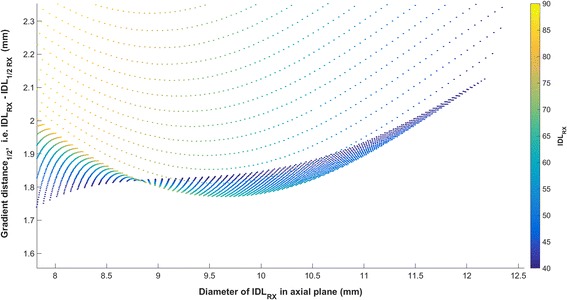
Overlap region where prescribing to a higher IDL maintains the same prescription isodose diameter in the axial plane but decreases the distance between the prescription and half-prescription line

Scatter plots of 10,251 shot within shot configurations are shown in Fig. 2a and b. For each plot, the gradient distance (factor = 0.5) in the axial dimensions is plotted versus the prescription isodose diameter. Figure 2a color codes each point by prescription IDL, while Fig. 2b color codes each point according to beam-on time. The latter calculation assumes a 20 Gy prescription, 2.5 Gy/min dose rate, and standard output factors of 0.8140 (4 mm) and 0.9005 (8 mm). As noted during the introduction, the prescription isodose diameter can be increased by decreasing the prescription IDL or increasing collimator size. This is clearly seen in the figures where two large jumps in the gradient distance represent transition zones moving from the 4–8 mm and 8–16 mm collimator settings. These transitions occur earlier when prescribing at higher IDLs. An overlap region exists around 8–11 mm where the gradient distance for plans prescribed at a higher IDL actually drops below the gradient distance for plans prescribed at a lower IDL. The region is expanded in Fig. 3 to show the overlap. Other areas of interest include the region prior to 6 mm where a higher IDL must be prescribed to match the diameter of smaller targets, and the region beyond 15 mm where the data converge. In comparing Fig. 2a and b for these regions, it can be seen that a marginal increase in the prescription IDL can reduce beam-on time while maintaining a sharp dose gradient. The reduction is nearly proportional to the ratio between two IDLs but slightly better knowing that prescribing to a higher IDL also means utilizing a higher weighting of the 8 mm and 16 mm collimator settings.

Figure 2a and b can also be used to visualize the optimization method described in section IIB. First, consider each figure as a grid where each location along the X axis defines a column. Only one plan can be chosen from within each column. The strategy used to minimize beam-on time for a given column is to first start with the Y location that represents the plan prescribed at the 50% IDL. From there all plans immediately above (within 3%) or below are considered, and the plan with the minimum beam-on time is chosen. An alternative optimization strategy would be to minimize the dose gradient by choosing the plan from within each column that has the smallest Y value. The two strategies produce similar results except within the transition zones where prescription IDLs less than 50% lead to markedly steeper gradients with associated increases in beam-on time and maximum target dose (see Additional file 1: Figure S5 and S6).

**Fig. 4:**
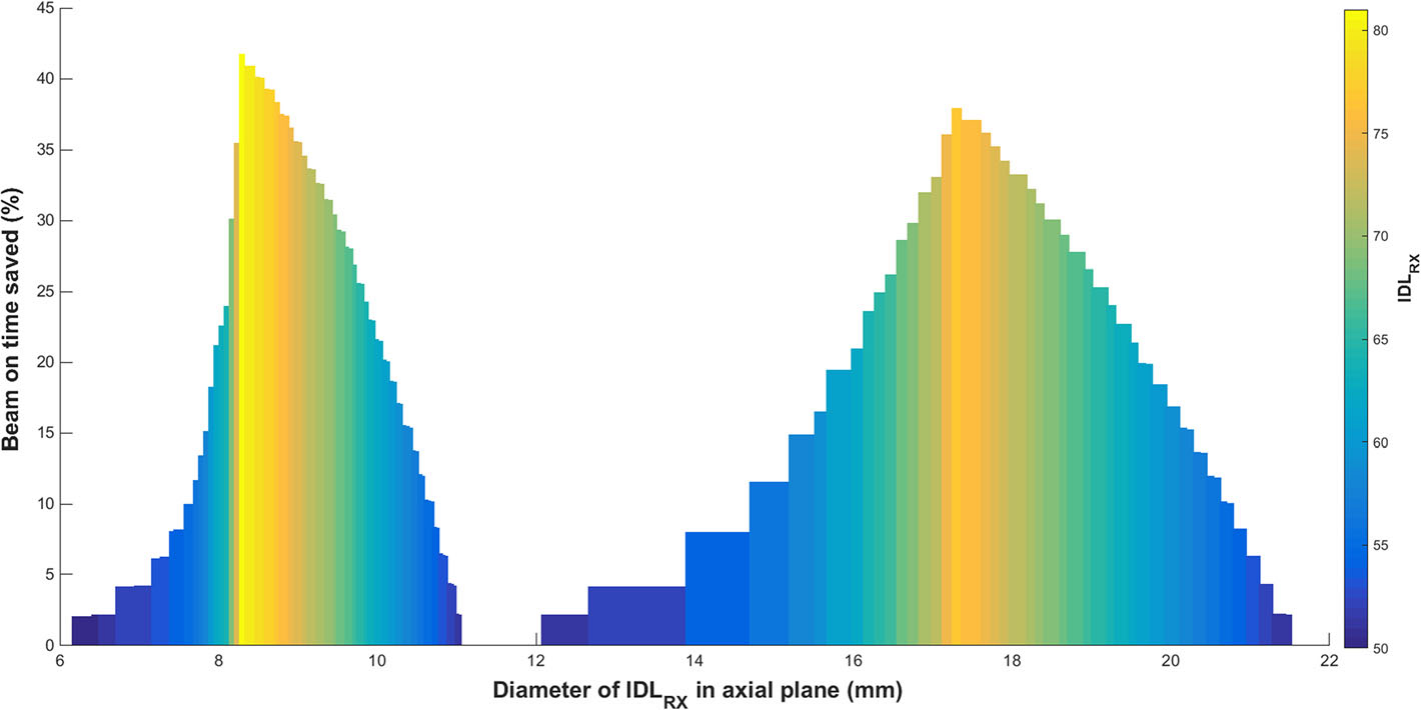
Twin peaks representing the time savings predicted when using shot within shot optimization. The comparison is made between shot within shot plans prescribed at the 50% IDL (SS_50%_) and shot within shot plans optimized using collimator weighting and different selections of the prescription IDL (SS_opt_). The optimal plan provided an equivalent diameter of the prescription IDL in the axial plane, a gradient distance (factor = 0.5) no worse than 3% from the original, and a minimized beam-on time

In order to quantify the reduction in beam-on time when applying shot within shot optimization, each configuration prescribed at the 50% IDL (SS_50%_ – 201 unique plans) was compared to the corresponding optimal configuration (SS_opt_ – again 201 unique plans) using both shot weighting and IDL optimization. One way to think of this is as a comparison between two plans within each column as described above. The optimal configuration provided an equivalent diameter of the prescription IDL in the axial plane, a gradient distance (factor = 0.5) no worse than 3% from the original, and a minimized beam-on time. The results are shown in Fig. 4 where the reduction in beam-on time can be visualized as twin peaks centered between 8 and 10 mm and 16–20 mm. The largest gains (~40%) occur immediately after the transition zones and reflect the use of prescription IDLs ranging from 50 to 80%. The size of the peaks can be altered by relaxing the similarity constraint for the gradient distance. A figure highlighting these changes can be found in the Additional file 1: Figure S7.

The optimization strategy was tested on seven patients previously treated for 1–4 brain metastasis (20 lesions total). Results are shown in Table 2 where a comparison is made between the actual treated plan, a re-plan using the shot within shot technique prescribed at the 50% IDL (SS_50%_), and a re-plan using the shot within shot technique prescribed at the optimal IDL (SS_opt_). The previously treated plans were prescribed almost exclusively to the 50% IDL and often involved the use of multiple shots with different stereotactic coordinates and composite sector weighting (14/20 treated with a multiple shots, 12/20 treated with multiple stereotactic coordinate locations). For the re-plans using the shot within shot technique, all shots maintained the same stereotactic coordinates and were weighted such that the same target coverage as the original plan was achieved. A complete description of the original plan settings and the shot weighting used for the re-plans can be found in the Additional file 2: patient data. For Table 2, the common GK metrics of selectivity and gradient index are included as well as the volume of normal brain receiving 12 Gy or above (V_12Gy_). The latter metric is widely used as a measure of radiation necrosis where the constraint is typically less than 5–10 cm^3^ [2, 3]. The reduction in beam-on time is shown in a comparison between the treated plan and the shot within shot re-plans (ΔT1), and in a comparison between the SS_50%_ plan and the optimized plan (ΔT2). In looking at the results, the optimization strategy reduced beam-on time by more than 15% in 16/20 lesions when compared to the treated plan, and in 12/20 lesions when compared to the SS_50%_ plan. For the latter comparison, the largest reductions were in line with expectations of ~40% (see Fig. 4), though the centers of the twin peaks were slightly shifted when plotted using an equivalent spherical diameter calculated from target volume (see Additional file 1: Figure S8). Remembering that the gradient distance was used as a similarity constraint, it is interesting to see how this metric tracks with the more familiar gradient index. Of the 20 lesions, 6 had a gradient index exceed a 3% difference, though only two were greater than 3.5%. The gradient index was actually smaller than the original plan in 3/20 lesions. In most cases the differences between V_12Gy_ were exceedingly small and were practically negligible when comparing the two shot within shot re-plans (max difference of 0.17 cm^3^).

**Table 1:** Description of terms and metrics used throughout the manuscript

Term/metric	Equation	Description
Dose gradient		General term referencing the rapid fall-off in dose along the target periphery.
Penumbra	$\left|{d}_{IDL_{80\%}}-{d}_{IDL_{20\%}}\right|$	The distance between two IDLs that lie within the dose gradient. Traditionally, the IDLs are chosen as the 80% and 20% lines.
Gradient distance	$\left|{d}_{IDL_{RX}}-{d}_{IDL_{RX}\times f}\right|$	Distance between two relative IDLs, where the first is the prescription IDL and the second is the prescription IDL multiplied by a factor ranging from 0.2–0.9.
Gradient index	$\frac{V_{1/2 RX}}{V_{RX}}$	Ratio of volume enclosed by half the prescription dose to that enclosed by the prescription dose.
Prescription isodose diameter	$\left|{d}_{Left,{IDL}_{RX}}-{d}_{Right,{IDL}_{RX}}\right|$	Diameter of the prescription isodose volume as visualized in the axial plane, i.e. distance between prescription IDLs located on the left and right side of the shot center.
Coverage	$\frac{V_{RX}\cap {V}_{target}}{V_{target}}$	Fraction of target volume within prescription isodose volume.
Conformity		General term referencing the degree to which the prescription isodose is contained within the target volume.
Selectivity	$\frac{V_{RX}\cap {V}_{target}}{V_{RX}}$	Fraction of prescription isodose volume within target volume. Also, the inverse of the conformity index multiplied by coverage.

## Discussion

In the first part of this work, the dose gradient was characterized for all three collimator settings available on the Gamma Knife Pefexion. The penumbra for this system has previously been reported, but only in terms of relative dose [4]. As GK plans are actually prescribed to IDLs within this region, the measurement provides little practical significance. By instead characterizing the gradient as a distance from the prescription IDL, the resulting data can be used to better assess the feasibility of certain treatments. Consider the following scenarios:

Scenario 1 – The treating physician would like to deliver 20 Gy to a lesion that lies 2 mm posterior to the brainstem. They would like to know if a maximum dose of 12 Gy to the brainstem can be achieved. In a best case scenario the answer is yes; using a factor of 0.6 and a prescription IDL of 50%, the distance to the 12 Gy line in the axial plane is 0.935 mm (4 mm collimator) or 1.336 mm (8 mm collimator).

Scenario 2 – The treating physician would like to deliver 14 Gy to a lesion 1 mm superior to the cochlea. They would like to know if a maximum dose of 4 Gy to the cochlea can be achieved. No, using a factor of 0.3 and a prescription IDL of 50%, the distance to the 14 Gy line in the sup/inf direction is 1.02 mm (4mm collimator) or 1.6 mm (8 mm collimator).

In the second part of this work, the data was expanded to incorporate the shot within shot technique. While this technique is familiar to GK users, it is surprisingly absent in the literature. With the introduction of sector collimation and automated couch movement, it is likely that users have migrated towards multi-shot, composite planning, particularly for asymmetric lesions and those that lie within close proximity to critical structures (meningioma, acoustic neuroma, pituitary adenoma, etc.) [5]. For brain metastases, however, a strong case can be made for the utilization of the shot within shot technique, even when lesions are not completely spherical. Two primary considerations for treating brain metastases located in non-eloquent regions are target coverage and V_12Gy_ of normal brain. As seen in Table 2, V_12Gy_ is already very small for the small to moderately sized metastases analyzed in this study. Using the shot within shot technique, these volumes were reduced in many cases, and in only one instance increased by more than 0.5 cm^3^. In terms of conformity, the selectivity was understandably better in many cases planned using multiple composite shots. However, this appears to be a tertiary concern when treating far from critical structures as conformity has been shown in previous studies to have no correlation with symptomatic radiation necrosis [2, 6, 7].

Going further, the shot within shot technique allows for optimization based on different selections of the prescription IDL and collimator weighting. For this work, beam-on time was selected as the parameter to minimize as it appeared to benefit the most from optimization. The method appears valid, particularly for lesions that fall within specific size ranges as seen in Fig. 4 and Additional file 1: Figure S8, and are not too peripheral in location (>1–2 cm from skull boundary based on visual assessment of phantom data). The reduction in beam-on time is real and represents value for all parties involved. From a patient standpoint, a faster delivery means less time spent in a potentially uncomfortable situation, and also less sedation if it is part of the treatment process. From a staffing standpoint, a reduction in beam-on time means less time at the machine for the authorized user and authorized medical physicist who must provide personal supervision per NRC regulations [8]. The treatment of multiple metastases can be lengthy, particularly on the GK as the Cobalt-60 sources decay. As such, the value of optimization is constantly increasing. Using patient case 4 as an example, the optimized plan is projected to reduce beam-on time from 78 to 44 min immediately post install (ΔT = 34 min), and 150 to 86 min at the 5 year mark (ΔT = 64 min).

In order to achieve the time savings observed in this work through the use of shot within shot optimization, there must be willingness to accept a decrease in the maximum target dose. There is evidence that this tradeoff is acceptable considering that both standard linac and robotic radiosurgery are commonly prescribed in the 65–90% IDL range without clear evidence of worsened outcomes [9–11]. A number of recent studies have further explored the rationale for GK based delivery. In one study, the authors found no association between the homogeneity index (max dose/peripheral dose) and local failure or radiation necrosis for 1–3 brain metastases treated with GK [12]. In a second study, the results actually suggested improved local control when prescribing at higher IDLs, particularly for small to moderately sized lesions [13]. With these studies in mind, the expectation when using shot within shot optimization is that neither local control nor radiation necrosis will be any worse than plans prescribed at the 50% IDL based upon similar target coverage and levels of V_12Gy_.

## Conclusion

Conventional wisdom assumes prescribing to the 50% IDL provides the steepest dose gradient for GK radiosurgery. The results of this study show that this is not always the case and that there are opportunities to increase the prescription IDL by using shot within shot optimization. In these cases beam-on times can be reduced by up to 40% while maintaining equivalent target coverage and V_12Gy_ of normal brain. Therefore, the proposed method can be used to reduce treatment times for patients without any expected decrement in tumor control or toxicity. Further research aims to prove this technique prospectively and compare to other GK planning strategies.

**Table 2:** Comparison between the actual treated plan (prescribed almost exclusively at the 50% IDL), a re-plan using the shot within shot technique prescribed at the 50% IDL (SS_50%_), and a re-plan using the shot within shot technique prescribed at the optimal IDL (SS_opt_)

Case	Vol (cc)	RX (Gy)	IDL (%)	Selectivity			Gradient index			Brain V_12Gy_ (cc)			ΔT1 (%)		ΔT2 (%)
			SS_opt_	Plan	SS_50%_	SS_opt_	Plan	SS_50%_	SS_opt_	Plan	SS_50%_	SS_opt_	Plan - SS_50%_	Plan - SS_opt_	SS_50%_ - SS_opt_
1A	0.21	21	65	0.67	0.51	0.50	3.50	2.62	2.63	0.68	0.69	0.71	35	53	28
2A	0.09	21	52	0.51	0.61	0.61	3.53	3.39	3.50	0.40	0.31	0.33	0	4	4
2A	0.10	21	51	0.31	0.58	0.58	2.78	3.42	3.45	0.64	0.37	0.38	0	0	0
2B	0.16	21	74	0.47	0.47	0.48	2.87	3.03	3.07	0.59	0.58	0.59	1	39	38
2C	1.13	18	65	0.72	0.72	0.67	3.20	3.10	2.96	2.00	1.99	2.16	48	61	25
3A	1.58	21	67	0.67	0.73	0.72	3.06	2.60	2.56	4.26	3.03	3.00	14	37	27
3B	0.16	21	58	0.67	0.74	0.73	3.17	3.22	3.31	0.45	0.41	0.44	1	15	16
3C	0.05	21	50	0.45	0.45	0.45	2.85	2.84	2.84	0.19	0.19	0.19	0	0	0
4A	0.18	21	76	0.64	0.58	0.67	3.63	2.84	3.08	0.62	0.52	0.50	29	56	38
4B	0.18	21	74	0.64	0.55	0.59	3.46	2.80	2.89	0.60	0.55	0.56	24	51	36
4C	0.11	21	63	0.71	0.50	0.52	3.58	3.17	3.39	0.34	0.46	0.49	16	35	23
4D	2.22	18	60	0.81	0.79	0.77	2.50	2.61	2.62	2.56	2.76	2.78	17	29	14
5A	1.40	20	75	0.74	0.60	0.61	3.17	2.55	2.52	3.20	3.18	3.03	62	76	37
5B	0.61	20	52	0.63	0.56	0.56	3.52	3.42	3.48	1.86	2.20	2.28	15	18	4
5C	0.04	20	57	0.37	0.22	0.22	3.17	3.26	3.40	0.25	0.46	0.44	19	30	14
6A	0.48	21	50	0.63	0.60	0.60	3.24	3.37	3.37	1.51	1.72	1.72	0	0	0
6B	0.83	21	56	0.68	0.54	0.56	2.99	2.95	3.08	2.20	2.94	2.92	46	52	11
6C	0.16	21	74	0.67	0.67	0.65	3.11	3.11	3.22	0.45	0.45	0.49	0	36	36
6D	0.11	21	77	0.40	0.40	0.38	2.91	2.91	2.89	0.58	0.58	0.61	0	40	40
7A	0.83	20	71	0.54	0.37	0.40	3.89	2.57	2.58	3.60	3.58	3.34	19	45	32

Reduction in beam-on times are shown in a comparison between the re-plans and the treated plan (ΔT1), and in comparison between the SS_50%_ plan and optimized plan (ΔT2)

## Additional files

Additional file 1: Figures S1. Multi-Gaussian fit of the data sampled from the planning system for the 8 mm collimator setting (X/Y axis above, Z axis below). Roughly 70 points were sampled for each collimator setting and direction using the line measurement tool available in the planning system. **Figure S2.** Dose gradient for the X/Y and Z dimensions, 16 mm collimator setting. **Figure S3.** Dose gradient for the X/Y and Z dimensions, 4 mm collimator setting. **Figure S4.** Gradient distance (factor = 0.5) in the axial plane when utilizing the shot within shot technique. **Figure S5.** Dose profiles in the axial dimension when using different shot within shot combinations to produce plans with the same prescription isodose diameter and similar dose gradients. The three numbers associated with each area plot are the weighting of the 4 mm, 8 mm, and 16 mm collimator settings. **Figure S6.** Curves representing shot within shot plans prescribed at the 50%IDL (blue) and those optimized for beam-on time (orange) and gradient distance (red). Notice the difference in the curves within the transition zones where prescribing to IDLs less than 50% minimizes the gradient distance. Because the optimization of beam-on time was designed to provide a similar gradient distance as plans prescribed at the 50% IDL, the blue and orange curves are very similar, though different in terms of beam-on time, prescription IDL, and maximum target dose. **Figure S7.** Twin peaks representing the time savings predicted when using shot within shot optimization. The different colors represent different similarity constraints for the gradient distance (factor = 0.5) ranging from 1 to 10%. **Figure S8.** Beam-on time saved using shot within shot optimization on 7 actual patients (20 lesions). The shape of the data is similar to that predicted based on phantom simulation. (DOCX 3977 kb)

Additional file 2: Profile validation and patient data. (XLSX 35 kb)

## Abbreviations

GK: Gamma Knife
IDL: Isodose line
SS_50%_: Re-plan using shot within shot technique prescribed at the 50% isodose line
SS_opt_: Re-plan using shot within shot technique prescribed at the optimized isodose line
V_12Gy_: Volume of normal brain receiving at least 12 Gy
ΔT1: Reduction in beam-on time in comparison to the original treated plan when using optimization
ΔT2: Reduction in beam-on time in comparison to the shot within shot plan prescribed at the 50% isodose line when using optimization
