# Supplementary material for: Optimization of the prescription isodose line for Gamma Knife radiosurgery using the shot within shot technique
Source: Radiat Oncol. 2017 Nov 25;12:187. doi: 10.1186/s13014-017-0919-4 (PMC5702131; doi:10.1186/s13014-017-0919-4)
Supplement: Supplementary file 1 — Multi-Gaussian fit of the data sampled from the planning system for the 8 mm collimator setting (X/Y axis above, Z axis below). Roughly 70 points were sampled for each collimator setting and direction using the line measurement tool available in the planning system. Figure S2. Dose gradient for the X/Y and Z dimensions, 16 mm collimator setting. Figure S3. Dose gradient for the X/Y and Z dimensions, 4 mm collimator setting. Figure S4. Gradient distance (factor = 0.5) in the axial plane when utilizing the shot within shot technique. Figure S5. Dose profiles in the axial dimension when using different shot within shot combinations to produce plans with the same prescription isodose diameter and similar dose gradients. The three numbers associated with each area plot are the weighting of the 4 mm, 8 mm, and 16 mm collimator settings. Figure S6. Curves representing shot within shot plans prescribed at the 50%IDL (blue) and those optimized for beam-on time (orange) and gradient distance (red). Notice the difference in the curves within the transition zones where prescribing to IDLs less than 50% minimizes the gradient distance. Because the optimization of beam-on time was designed to provide a similar gradient distance as plans prescribed at the 50% IDL, the blue and orange curves are very similar, though different in terms of beam-on time, prescription IDL, and maximum target dose. Figure S7. Twin peaks representing the time savings predicted when using shot within shot optimization. The different colors represent different similarity constraints for the gradient distance (factor = 0.5) ranging from 1 to 10%. Figure S8. Beam-on time saved using shot within shot optimization on 7 actual patients (20 lesions). The shape of the data is similar to that predicted based on phantom simulation. (DOCX 3977 kb) [file 13014_2017_919_MOESM1_ESM.docx]

**Additional file 1**

**
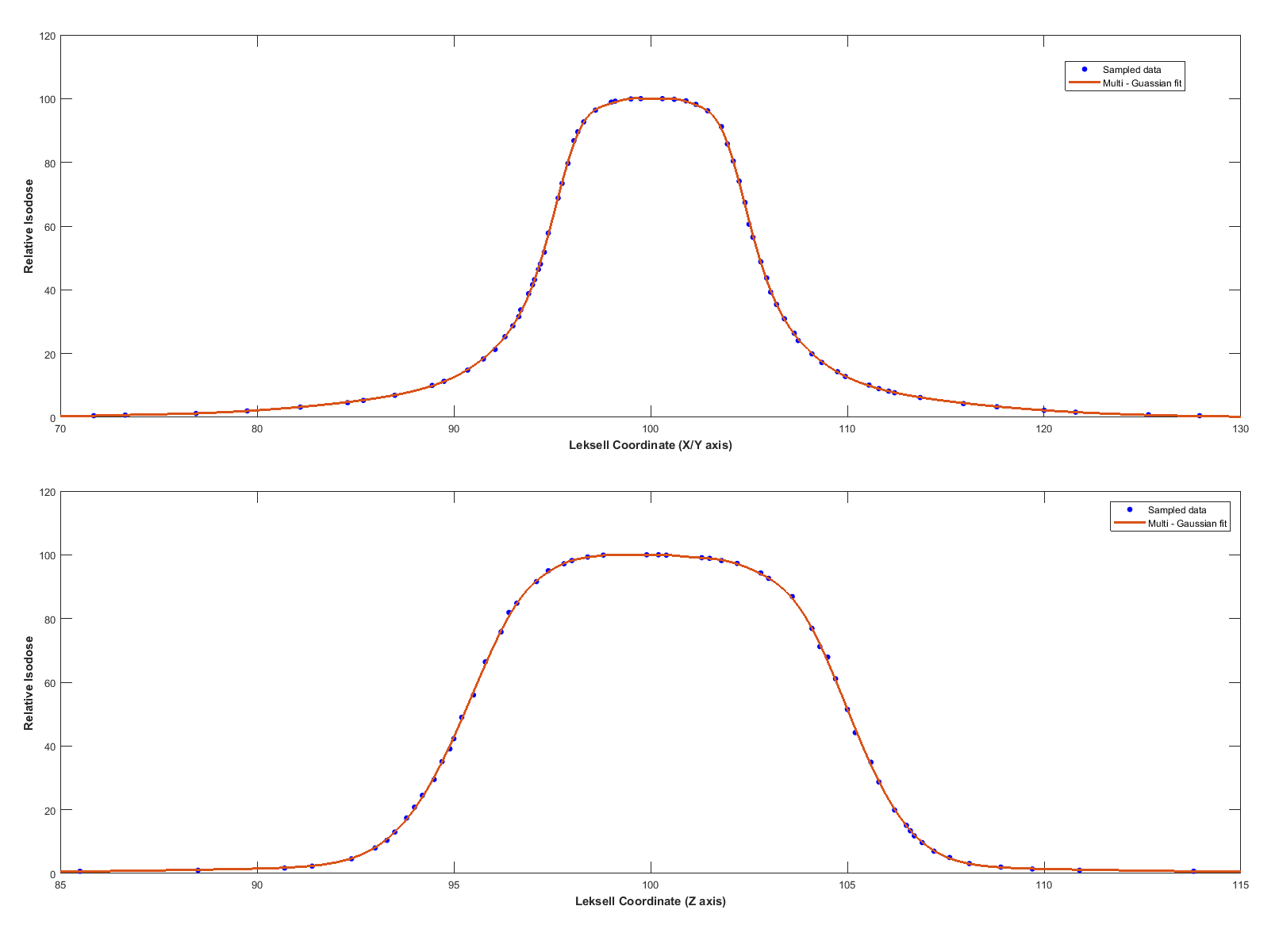
**

**Figure S1.** Multi-Gaussian fit of the data sampled from the planning system for the 8 mm collimator setting (X/Y axis above, Z axis below). Roughly 70 points were sampled for each collimator setting and direction using the line measurement tool available in the planning system.


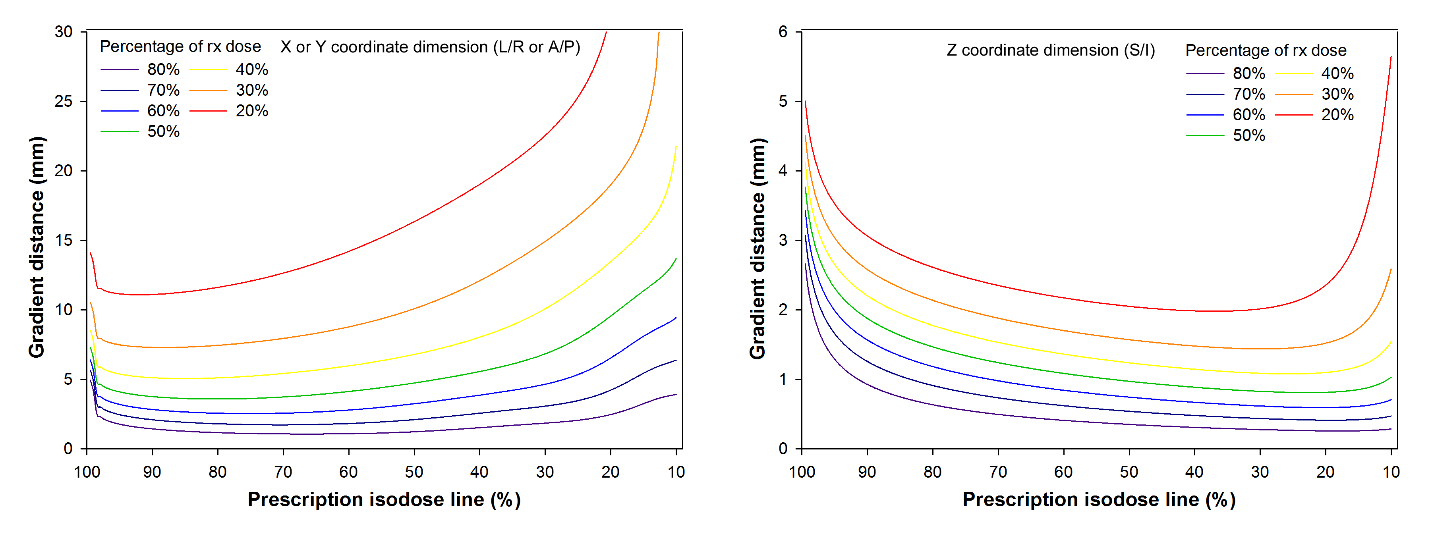


**Figure S2.** Dose gradient for the X/Y and Z dimensions, 16 mm collimator setting.


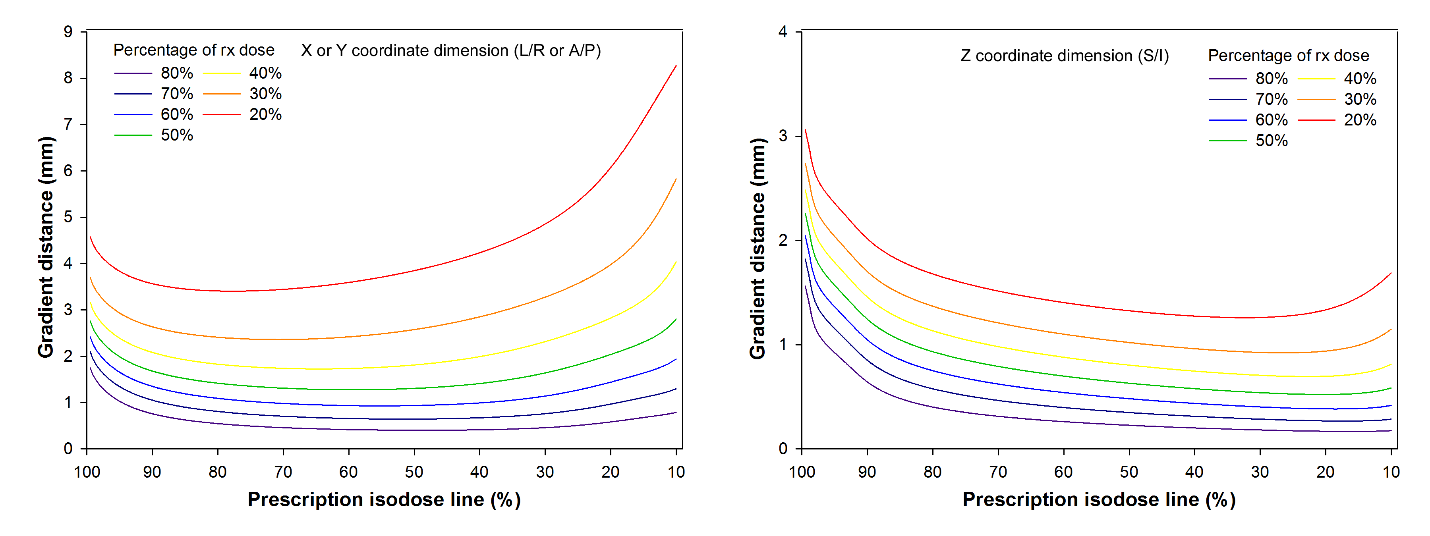


**Figure S3.** Dose gradient for the X/Y and Z dimensions, 4 mm collimator setting.


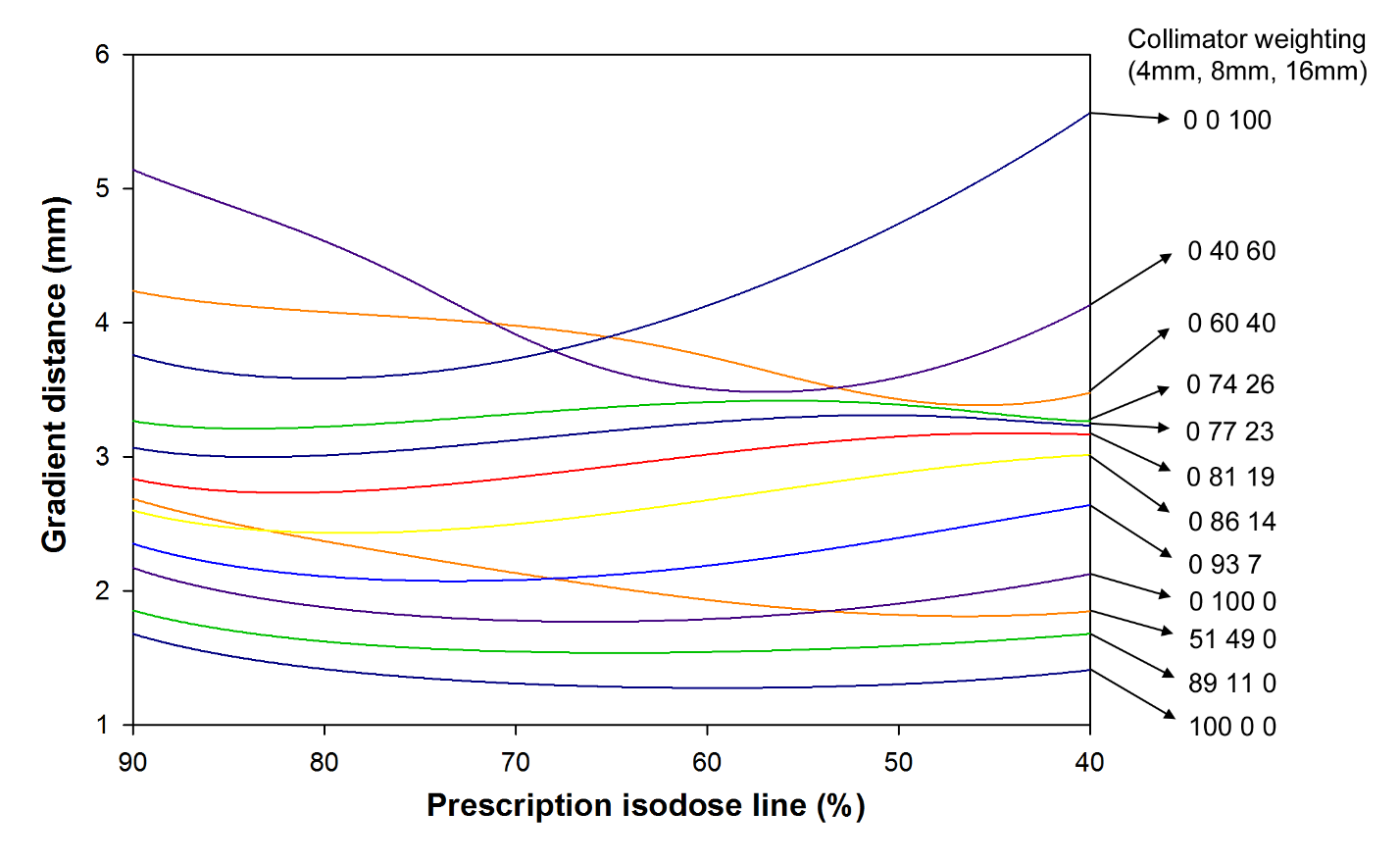


**Figure S4.** Gradient distance (factor = 0.5) in the axial plan when utilizing the shot within shot technique.


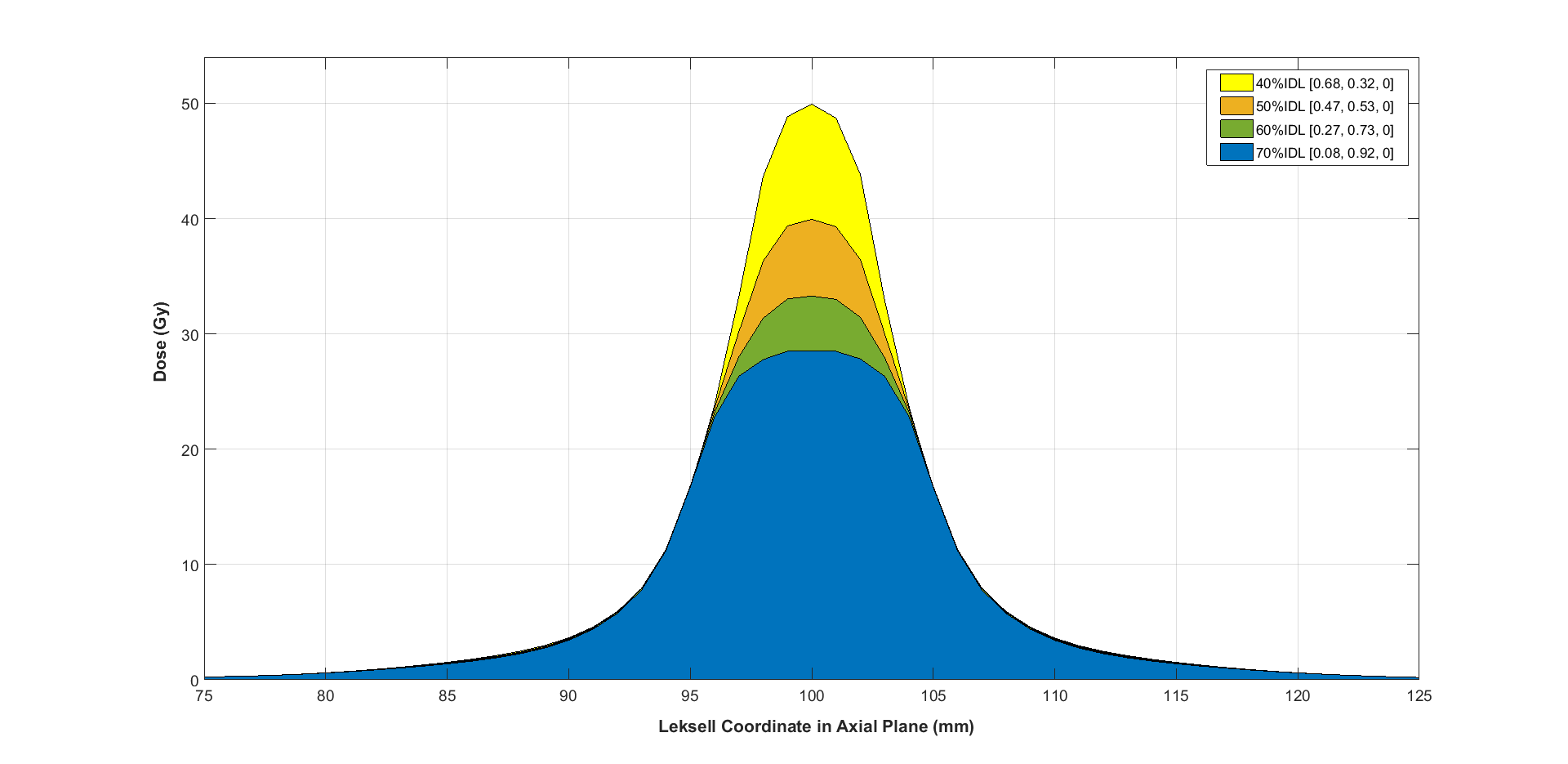


**Figure S5.** Dose profiles in the axial dimension when using different shot within shot combinations to produce plans with the same prescription isodose diameter and similar dose gradients. The three numbers associated with each area plot are the weighting of the 4 mm, 8 mm, and 16 mm collimator settings.


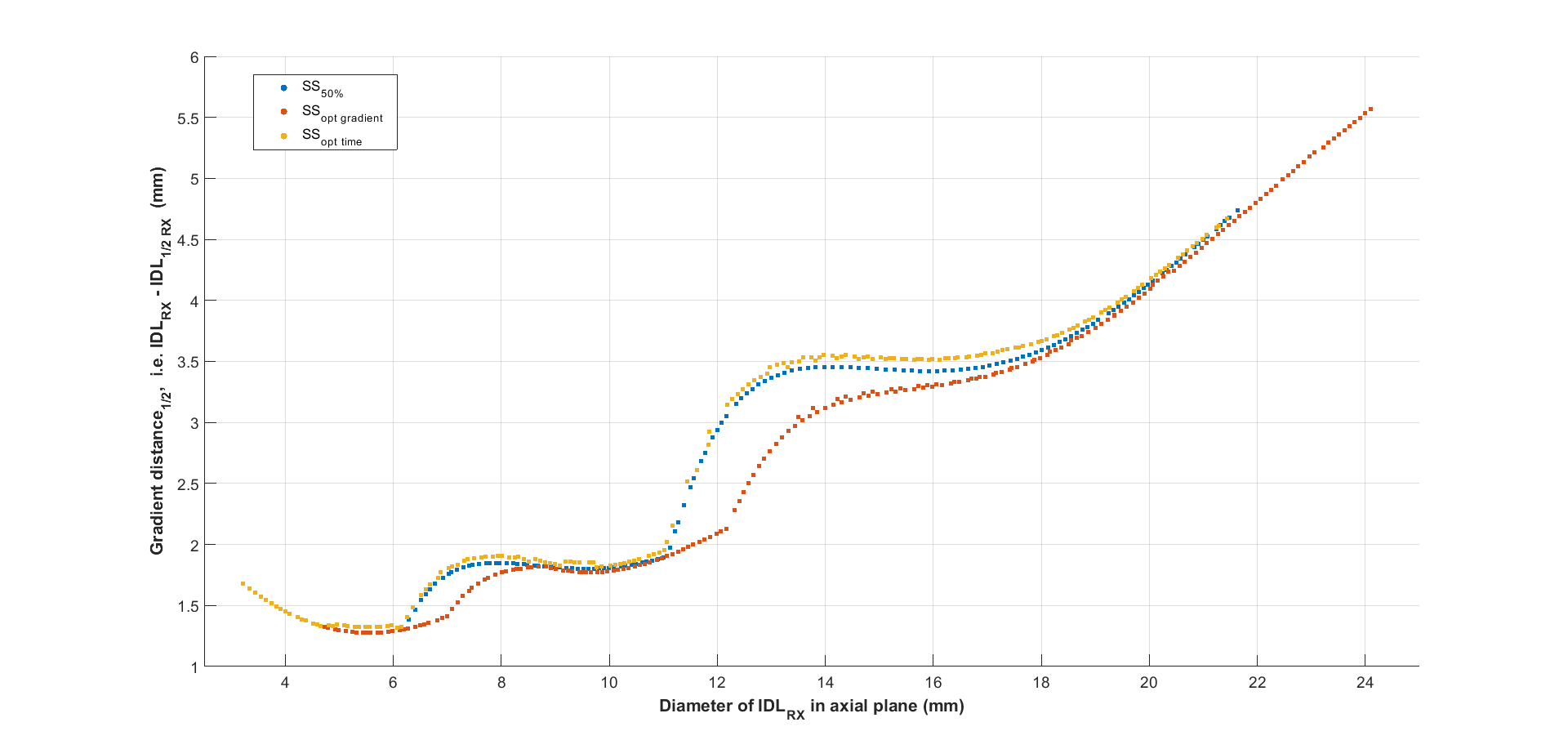


**Figure S6.** Curves representing shot within shot plans prescribed at the 50%IDL (blue) and those optimized for beam-on time (orange) and gradient distance (red). Notice the difference in the curves within the transition zones where prescribing to IDLs less than 50% minimizes the gradient distance. Because the optimization of beam-on time was designed to provide a similar gradient distance as plans prescribed at the 50% IDL, the blue and orange curves are very similar, though different in terms of beam-on time, prescription IDL, and maximum target dose.


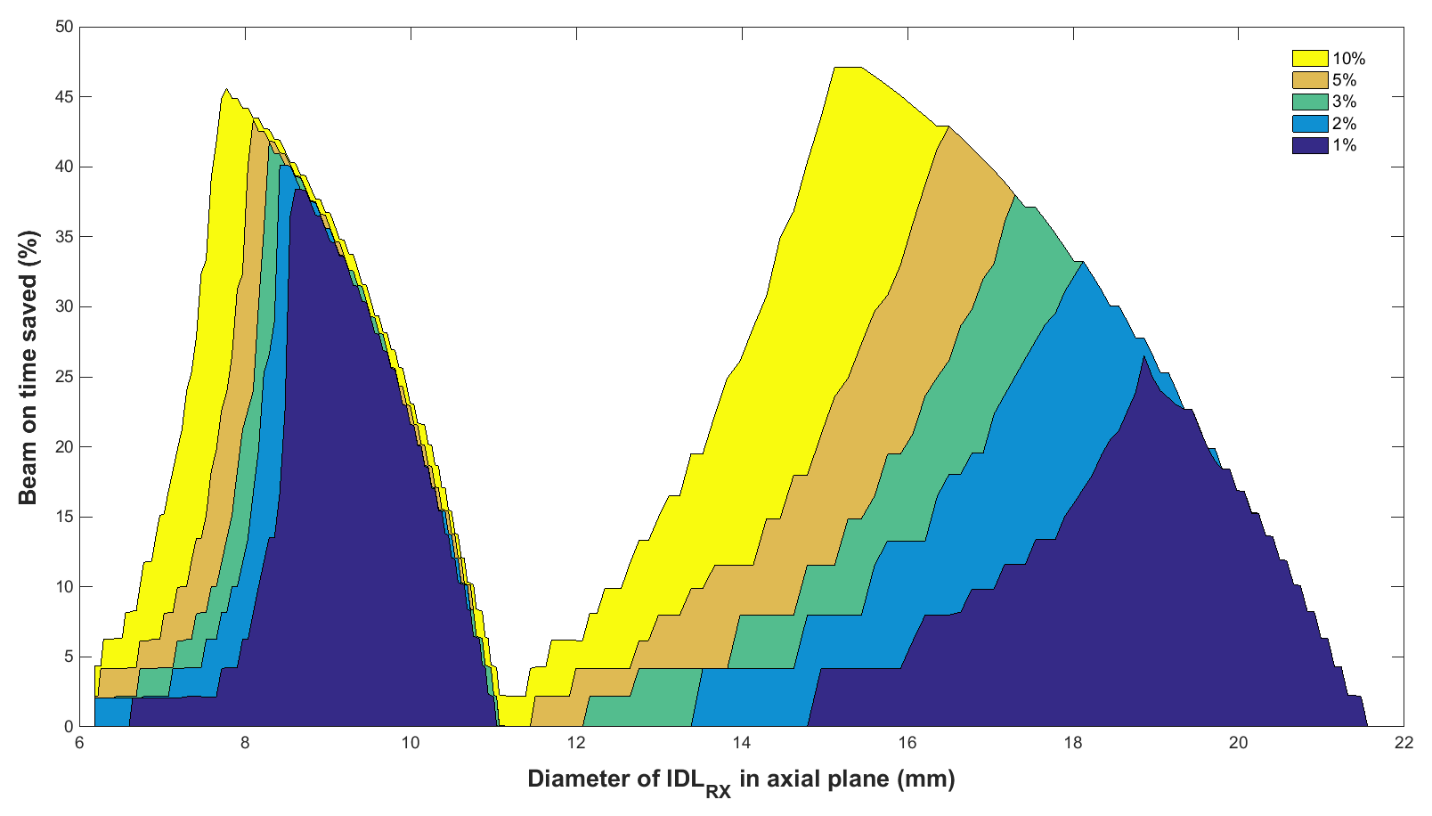


**Figure S7.** Twin peaks representing the time savings predicted when using shot within shot optimization. The different colors represent different similarity constraints for gradient distance factor=0.5 ranging from 1 – 10%.


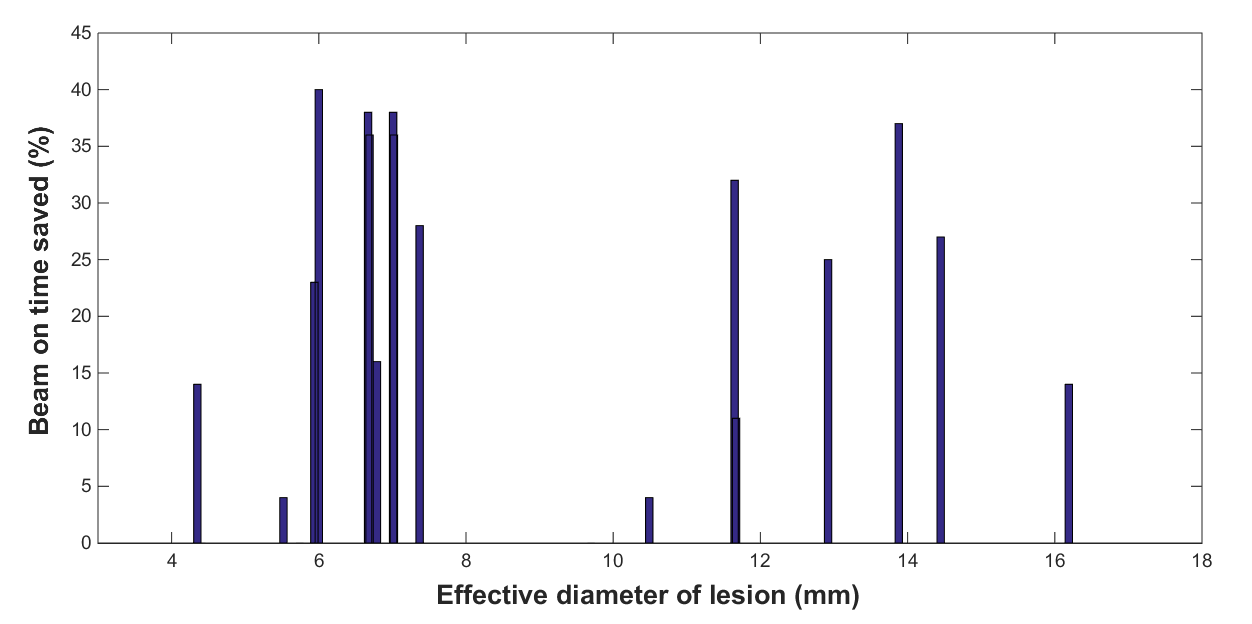


**Figure S8.** Beam-on time saved using shot within shot optimization on 7 actual patients (20 lesions). The shape of the data is similar to that predicted based on phantom simulation.
